# Molecular phylogeny reveals cryptic diversity in *Sibynophis* from China (Serpentes: Sibynophiidae)

**DOI:** 10.1002/ece3.10367

**Published:** 2023-07-28

**Authors:** Peng Guo, Ping Wang, Bing Lyu, Qin Liu, Jieyu Zheng, Chunmei Fu, Yayong Wu, Guocheng Shu, Shaobing Hou

**Affiliations:** ^1^ Faculty of Agriculture, Forest and Food Engineering Yibin University Yibin China; ^2^ Sichuan Academy of Forestry Chengdu China; ^3^ State Key Laboratory of Genetic Resources and Evolution & Yunnan Key Laboratory of Biodiversity and Ecological Conservation of Gaoligong Mountain Kunming Institute of Zoology, Chinese Academy of Sciences Kunming China

**Keywords:** Asia, distribution, *Sibynophis grahami*, snakes, taxonomy

## Abstract

The elucidation of species diversity and distribution is critical within the fields of evolution, genetics, and conservation. The genus *Sibynophis* contains rare snakes that have historically received little attention. In this study, we conducted comprehensive sampling and use both mitochondrial and nuclear genetic markers to explore *Sibynophis* species diversity within China. Our findings revealed that *S. c. miyiensis* should be considered synonymous with *S. c. grahami*, and *S. c. grahami* should be gave a specific rank as *S. grahami*. In addition, we discovered *S. triangularis* was new to China and Myanmar. Based on the specimens and molecular phylogeny results, we redefined the species distribution boundaries of each Chinese species.

## Graphical abstract

This study first explored the species diversity of Chinese species of the genus *Sibynophis* using both mitchondiral and nuclear gene data. The results indicated that *S. c. miyiensis* is a synonym of *S. c. grahami*, and *S. c. grahami* should be gave a specific rank as *S. grahami*. *Sibynophis triangularis* was uncovered to be new to China and Myanmar. The species distribution boundaries of each Chinese species were redefined.

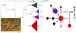

## 1 INTRODUCTION

Species diversity is central to biodiversity. Exploration of species diversity and identification of species distribution boundaries are pivotal to the preservation and management of biodiversity. The accumulation of multilocus data and development of related analytical technologies have greatly advanced our understanding of cryptic species diversity. In recent decades, considerable progress has been made in the phylogeny of Asian snakes. Notably, various systematic issues have been addressed (Guo et al., 2020; Hou et al., 2021; Liu et al., 2021; Poyarkov et al., 2022), and an increasing number of new taxa have been described (Hou et al., 2021; Liu et al., 2018; Ren et al., 2022). However, in comparison with well‐studied common snake groups, such as *Hebius* and *Lycodon* (Guo et al., 2013, 2014; Hou et al., 2021; Ren et al., 2022), little attention has been paid to rare snake species, such as those in the genus *Sibynophis*.

The family Sibynophiidae is comprised of only two genera, including the nonvenomous genus *Sibynophis*. These snakes are characterized by their small body size, loose attachment of their dentary to the articular bone, and the presence of numerous small maxillary teeth of uniform size (Pope, 1935). Currently, nine species are recognized within *Sibynophis*, with primary distribution in South and Southeast Asia (Uetz et al., 2022; Wallach et al., 2014). Two species, *S. collaris* and *S. chinensis*, are known to occur in China (Wang et al., 2020; Zhao, 2006). *Sibynophis chinensis* was initially described based on a sole specimen from Yichang, Hubei, China (Günther, 1889), but is now known to occur in China, Korea, and Vietnam (Wallach et al., 2014). In China, the species exhibits broad distribution, ranging from southern Xizang in the west to Jiangsu in the east (Zhao, 2006). Three subspecies are currently recognized (Zhao, 2006; Zhao et al., 1998), including *S. c. chinensis*, *S. c. grahami*, and *S. c. miyiensis*. In addition to the nominal species, which broadly occurs in Southwest, Central, and East China, *S. c. miyiensis* is endemic to Southwest Sichuan and Northwest Yunnan, while *S. c. grahami* is endemic to northeastern Yunnan and Guizhou (Zhao et al., 1998; Zhao & Yang, 1997). This taxonomical arrangement is followed by most authors (Uetz et al., 2022; Wallach et al., 2014; Wang et al., 2020). The type locality of *S. collaris* is in Assam, India (Gray, 1853), but the species is restricted to Xizang and Yunnan in China (Zhao, 2006; Zhao et al., 1998). Morphologically, the two species differ from one another by number of supralabials and anterior temporal scales only (Zhao, 2006; Zhao et al., 1998).

Due to their rare occurrence and elusive nature, few *Sibynophis* specimens have been collected since their initial description, and few studies have been conducted on their biology, particularly their systematics (Li et al., 2020; Pyron et al., 2013; Zaher et al., 2019). Although *Sibynophis* species have been included in certain phylogenetic studies (Chen et al., 2013; Li et al., 2020; Pyron et al., 2013; Zaher et al., 2019), only a limited number of samples have been examined. For example, Zaher et al. (2019) conducted a large‐scale molecular phylogenetic study of advanced caenophidian snakes, which included five *Sibynophis* species, each with only one individual. Thus, given the small number of species and specimens studied, the diversity and evolution of the genus remain poorly understood.

Here, based on more extensive samples, we explored species diversity of the genus *Sibynophis* and revised the taxonomy and distribution boundaries of each taxon within China.

## 2 MATERIALS AND METHODS

**FIGURE 1:**
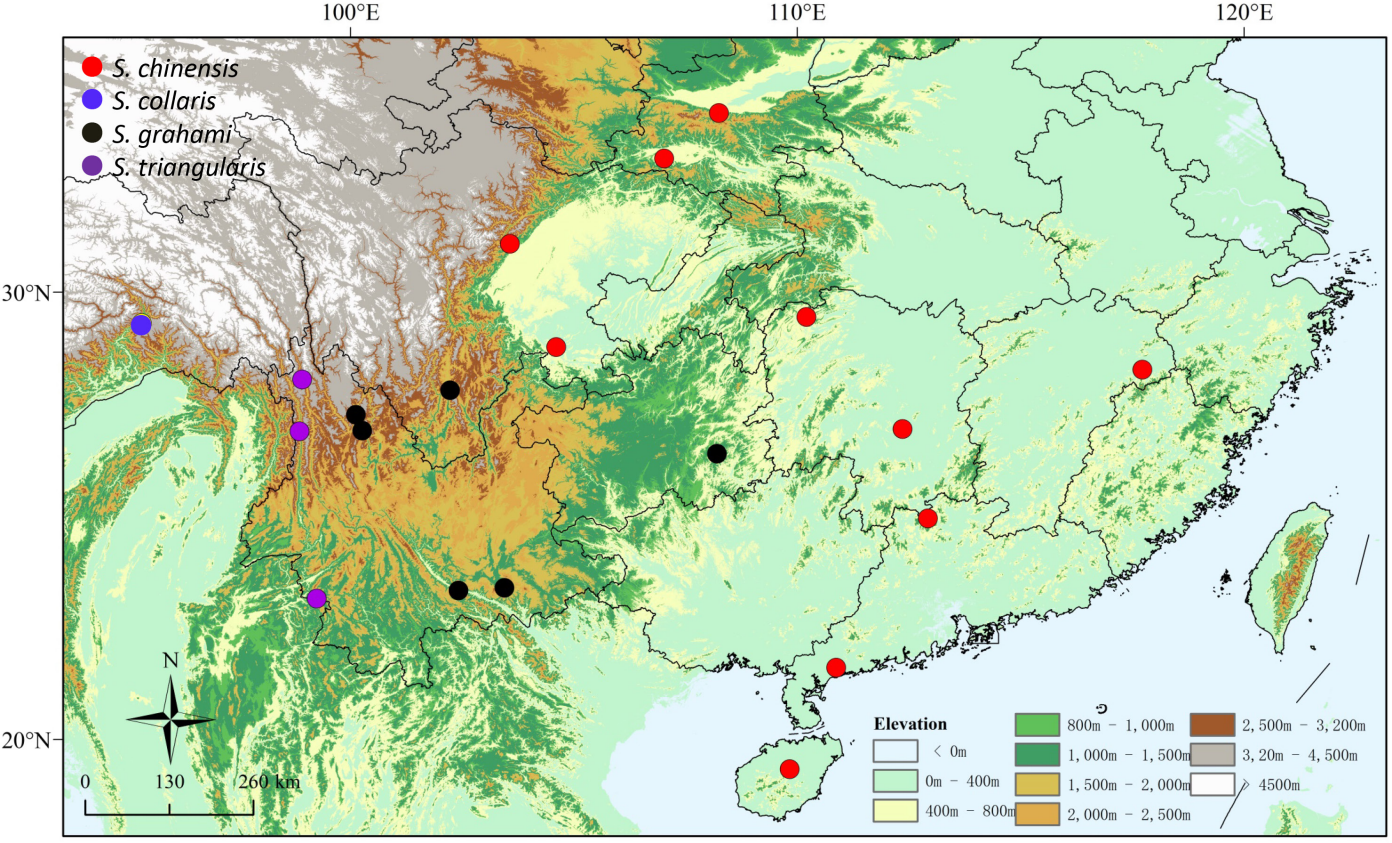
Map showing localities of Chinese samples of *Sibynophis* used in this study.

In total, 24 specimens previously identified as *S. collaris* and *S. chinensis* were collected from China (Figure 1), sequenced, and analyzed. Additional samples from the two species and their congeners were included, and their sequences were retrieved from GenBank (Table 1). *Scaphiodontophis annulatus* was chosen as the outgroup based on previous work (Pyron et al., 2013).

**TABLE 1:** Information for samples used in this study.

Taxon	Sample no.	Locality	Accession no.		
			cyt. *b*	ND2	c‐mos
*Sibynophis bistrigatus*	CAS 214081	Mandalay, Myanmar	KC000127	KC000130	KC000112
*Sibynophis chinensis*	CHS 695	Badagongshan, Hunan, China	–	MK199034	–
*S. chinensis*	CHS 753	Dawuling, Guangdong, China	MK201506	MK199065	–
*S. chinensis*	CHS 245	Mangshan, Hunan, China	MK201363	MK198941	–
*S. chinensis*	CHS 246	Heihe, Shaanxi, China	MK201364	MK198942	–
*S. chinensis*	BBRB090907‐1	Jeju Island, Korea	KF360246	KF360246	–
*S. chinensis*	CHS 2010075	Hanzhong, Shaanxi, China	KC000124	KC000131	KC000113
*S. chinensis*	ROM 25618	Ha Tay, Vietnam	MW199783	–	KX694818
*S. chinensis*	ROM 35661	Cao Bang, Vietnam	MW199781	–	MW177794
*S. chinensis*	KIZ 09100	Hainan, China	MW199782	OQ981580	MW177795
*S. chinensis*	KIZ 022270	–	OQ981557	–	–
*S. chinensis*	YBU18168/GP5499	Dujiangyan, Sichuan, China	OQ981568	OQ981587	OQ981601
*S. chinensis*	YBU18167/GP5498	Dujiangyan, Sichuan, China	OQ981567	OQ981586	OQ981600
*S. chinensis*	YBU11224/GP1960	Leigongshan, Guizhou, China	OQ981564	OQ981583	OQ981592
*S. chinensis*	YBU13274/GP2906	Hengyang, Hunan, China	OQ981565	OQ981584	OQ981598
*S. chinensis*	GP3673	Qianshan, Jiangxi, China	OQ981553	OQ981573	OQ981599
*S. chinensis*	GP1761	Hainan, China	OQ981550	OQ981571	OQ981597
*S. chinensis*	GP846	China	OQ981555	OQ981576	OQ981602
*S. chinensis*	GP 10550	Yibin, Sichuan, China	OQ981548	OQ981570	OQ981596
*Sibynophis collaris*	CAS 220133	Chin State, Myanmar	KC000129	KC000138	KC000117
*S. collaris*	CHS 879	Motuo, Linzhi, Tibet, China	MK201583	MK199136	–
*S. collaris*	CHS 880	Motuo, Linzhi, Tibet, China	–	MK199137	–
*S. collaris*	CAS 243217	Chin State, Myanmar	KC000125	KC000136	KC000114
*S. collaris*	CAS 234907	Chin State, Myanmar	KC000121	KC000135	KC000118
*S. collaris*	CAS 240150	Magway, Myanmar	KC000126	KC000134	KC000119
*S. collaris*	KIZ 011001	Motuo, Tibet, China	MW199784	OQ981577	MW177797
*S. collaris*	KIZ 07349	Motuo, Tibet, China	MW199785	OQ981579	MW177798
*S. collaris*	KIZ 06633	Motuo, Tibet, China	OQ981561	–	–
*S. collaris*	JN211315	–	JN211315	JN211315	–
*S. collaris*	GP2034	Motuo, Xizang, China	OQ981551	–	–
*S. collaris*	GP5665	Motuo, Xizang, China	–	OQ981575	OQ981594
*Sibynophis grahami*	YBU11042/GP1615	Southern Yunnan, China	OQ981562	OQ981581	OQ981590
*S. grahami*	CHS 244	Honghe, Yunnan, China	MK201362	–	–
*S. grahami*	KIZ 011982 /YPX18128	Mengzi, Yunnan, China	MW199787	OQ981578	MW177800
*S. grahami*	GP3649	China	OQ981552	OQ981572	OQ981593
*S. grahami*	GP1662	China	OQ981549	–	–
*S. grahami*	YBU 11043/GP1616	Southern Yunnan, China	OQ981563	OQ981582	OQ981591
*S. grahami*	YBU 23188/GP10544	Yulong, Yunnan, China	OQ981569	OQ981588	OQ981589
*S. grahami*	YBU15152/GP4142	Xichang, Sichuan, China	OQ981566	OQ981585	–
*S. grahami*	KIZ 028322/YPX 53026	Sanglila, Yunnan, China	OQ981556	–	–
*Sibynophis subpunctatus*	RAP0491		KC347471	–	KC347411
*Sibynophis triangularis*	FMNH 263023	Mondolkiri, Cambodia	KC000123	KC000132	KC000116
*S. triangularis*	CAS 214974	Nujiang, Yunnan, China	KC000128	KC000137	KC000115
*S. triangularis*	CAS 240616	Mon State, Myanmar	KC000122	KC000133	KC000120
*S. triangularis*	ROM 35660	Cao Bang, Vietnam	MW199786	–	MW177799
*S. triangularis*	KIZ 038285	Fugong, Yunnan, China	OQ981558	–	–
*S. triangularis*	KIZ 038286	Fugong, Yunnan, China	OQ981559	–	–
*S. triangularis*	KIZ 038287	Fugong, Yunnan, China	OQ981560	–	–
*S. triangularis*	GP4700	Cangyuan, Yunnan, China	OQ981554	OQ981574	OQ981595
*Scaphiodontophis annulatus*	KU 289943		GQ927323	–	–

Abbreviations: CAS, California Academy of Science, San Francisco; GP, Guo Peng, own catalog number; KIZ, Kunming Institute of Zoology, the Chinese Academy of Sciences; ROM, Royal Ontario Museum, Toronto; YBU, Yibin University, China.

Total DNA was extracted from 85% alcohol‐preserved liver or muscle tissues using M5 HiPer Universal DNA Mini Kit (Mei5 Biotechnology Co., Ltd.) following the manufacturer's protocols. Two mitochondrial gene fragments, cytochrome *b* (cyt. *b*) and NADH subunit 2 (ND2), and nuclear gene oocyte maturation factor mos (c‐mos), were amplified by polymerase chain reaction (PCR) using primers L14910/H16064 (Burbrink et al., 2000), ND2L49/ND2H50 (Alfaro & Arnold, 2001), and S77/S78 (Lawson et al., 2005). Cycling parameters were identical to those described in the above studies. Prior to sequencing, PCR products were purified using various commercial kits. The double‐stranded product was sequenced by a commercial company (GENEWIZ Company).

The sequences were manually edited using SeqMan in Lasergene v15.1 (DNASTAR Inc.), aligned using Muscle with default settings, and quality‐checked using MEGA v7.0 (Kumar et al., 2008; Tamura et al., 2013). Phylogenetic analyses were performed based on the two mitochondrial DNA (mtDNA) fragments as well as the combined data of mtDNA and nDNA using Bayesian inference (BI) and maximum‐likelihood (ML). Bayesian inference was executed in MrBayes v3.2.2 (Ronquist et al., 2012) using evolutionary models selected in PartitionFinder v2.1.1 under Bayesian information criterion (BIC) (Lanfear et al., 2012). All searches were performed with three independent runs, each initiating a random tree. Each run consisted of four Markov chains (three heated and one cold chain), with 2 × 10^6^ generations, sampling every 1000 generations, and 25% of initial samples discarded as burn‐in. Convergence was assessed by examining effective sample size (ESS) (>200) and likelihood plots through time in Tracer v1.7 (Rambaut et al., 2018). The resulting trees were combined to calculate posterior probabilities (PP) for each node in a 50% majority‐rule consensus tree. The ML trees were constructed using RAxML v7.2.6 (Stamatakis, 2006) with the GTMMAGTRCAT model under the same partitioning scheme as BI analysis. Branch support was assessed using 1000 nonparametric bootstrap (BS) topological replicates.

We constructed a haplotype network to depict interspecific/clade relationships based on c‐mos sequences. Analyses were executed using PopART v1.7 (Leigh & Bryant, 2015) with the parameter epsilon set to 0.

Pairwise genetic distances (*p*‐distances) between species or mtDNA clades were also calculated using Mega v7.0 (Kumar et al., 2008; Tamura et al., 2013).

## 3 RESULTS

**FIGURE 2:**
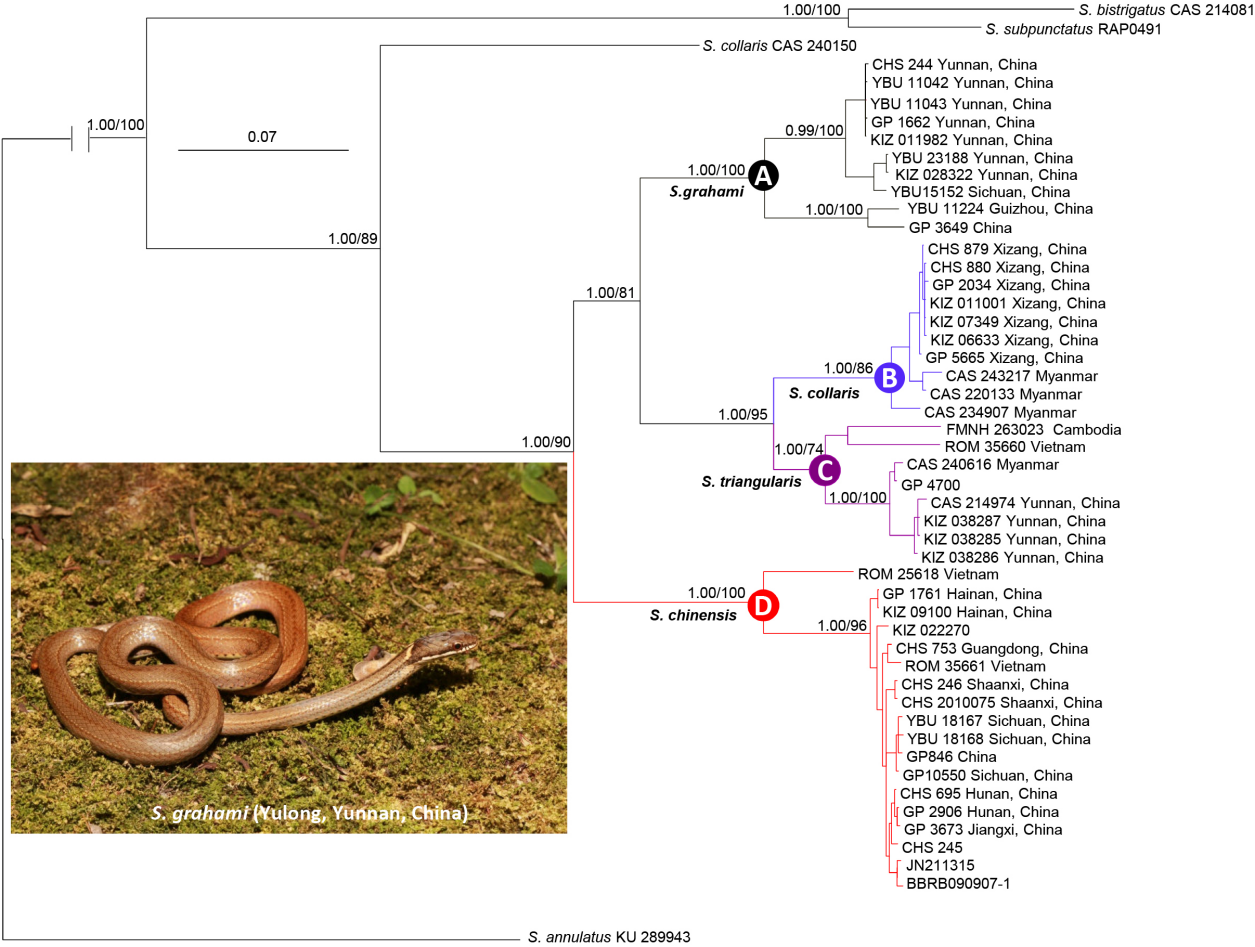
Bayesian consensus tree of *Sibynophis* inferred from mitochondrial DNA using models detailed in text. Posterior probabilities (>0.50) and bootstrap support values (>50%) are given adjacent to respective nodes for major nodes. Branch support indices are not given for shallow nodes to preserve clarity.

The best‐fit evolutionary models of the data were: HKY + G for the first coding position of cyt. *b* and ND2, HKY + I for the second position of cyt. *b* and ND2, the first and the third position of c‐mos, GTR + G for the third position of cyt. *b* and ND2, and K81UF for the second position of c‐mos. The BI and ML analyses based on mtDNA depicted relatively consistent topologies (Figure 2), and phylogenetic results based on the combined data were also mostly congruent with these of mtDNA‐based with an exception of *Sibynophis triangularis*, which is not a monophyletic group (data not shown). Phylogeny analyses based on mtDNA indicated all species of *Sibynophis* formed a highly supported lineage. Sister species *S. bistrigatus* and *S. subpunctatus* diverged first from the remaining members of the lineage, while all other specimens formed another monophyletic lineage with strong support. The Chinese specimens were positioned in four distinct but highly supported clades (A–D). Clade A was composed of specimens from eastern Yunnan and Guizhou, China; clade B consisted of samples from China (Xizang) and Myanmar; clade C contained specimens from China (Yunnan), Vietnam, Myanmar, and Cambodia; and clade D contained specimens from Vietnam and China (Sichuan, Shaanxi, and southern China). The four clades formed a highly supported monophyletic group with well‐resolved interclade relationships (D (A, (B, C))). A sample previously identified as *S. collaris* (CAS 240150) is much distinct from the other *S. collaris* identified samples (B clade).

Uncorrected *p*‐distances between species and clades are listed in Table 2. The interspecific/clade genetic distances ranged from 8.3% (clades B and C) to 21.2% (clade B and *S. bistrigatus*) based on cyt. *b* and from 7.9% (clades B and C) to 18.3% (clade D and sample CAS 240150) based on ND2 (Table 2).

**TABLE 2:** Genetic distance (*p*‐distance, %) based on two mtDNA fragments (cyt. *b*/ND2).

Taxa/clade	*S. bistrigatus*	*S. subpunctatus*	A (*S. grahami*)	B (*S. collaris*)	C (*S. triangularis*)	D (*S. chinensis*)	*S. collaris* (CAS 240150)
*S. bistrigatus*		9.4	20.2	21.2	20.8	20.0	19.6
*S. subpunctatus*	–		20.2	20.9	19.9	19.7	19.9
A (*S. grahami*)	36.1	–		12.2	12.3	12.6	16.1
B (*S. collaris*)	32.7	–	13.3		8.3	13.9	16.8
C (*S. triangularis*)	25.4	–	11.9	7.9		14.1	15.9
D (*S. chinensis*)	25.0	–	14.9	14.0	14.7		15.1
*S. collaris* (CAS 240150)	24.5	–	15.6	15.6	17.7	18.3	

Abbreviations: *S. bistrigatus*, *Sibynophis bistrigatus*; *S. chinensis*, *Sibynophis chinensis*; *S. collaris*, *Sibynophis collaris*; *S. grahami*, *Sibynophis grahami*; *S. subpunctatus*, *Sibynophis subpunctatus*; *S. triangularis*, *Sibynophis triangularis*.

**FIGURE 3:**
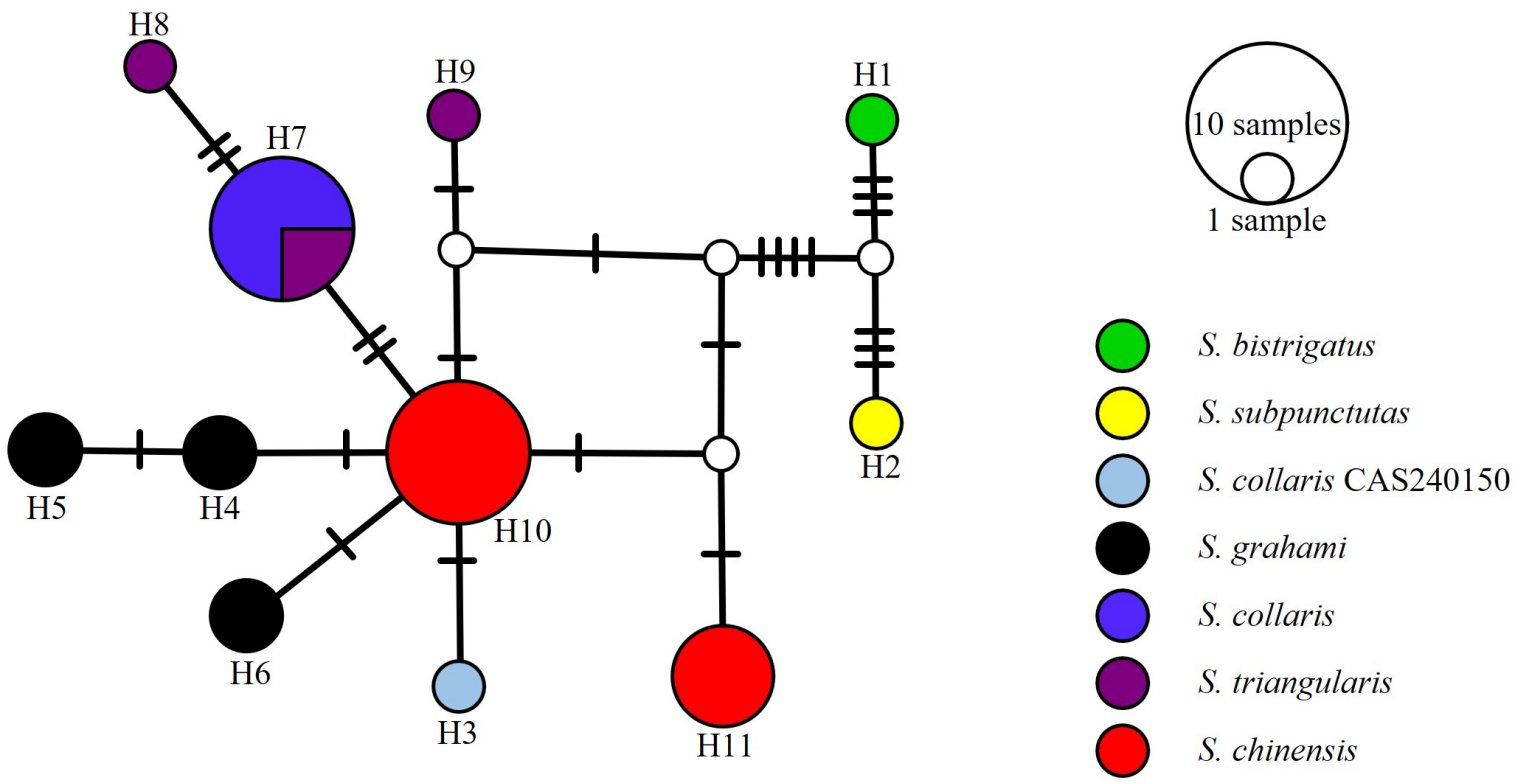
The network based on c‐mos gene (lines between the haplotypes represent one mutational step).

The network inferred from the c‐mos gene showed each species/clade exhibited unique haplotype, except clade B (*S. collaris*), which shared a haplotype with clade C (*S. triangularis*). *Sibynophis c. grahami*, *S. c. chinensis*, and *S. triangularis* contained three, two, and two unique haplotypes, respectively (Figure 3).

## 4 DISCUSSION

This study represents the first comprehensive investigation of *Sibynophis* systematics and diversity in China. The phylogenetic relationships reconstructed using the two methods and two datasets generated mostly identical topologies and highly similar support indices for major clades, with slight differences in support for several shallow nodes. Phylogenetic analyses further revealed that Chinese species of *Sibynophis* consisted of four clades (A–D), with well‐supported interclade relationships. Network analyses showed similar result.

### 4.1 Systematics of *S. chinensis*

*Sibynophis chinensis* is widely distributed in China. Here, the mtDNA‐based phylogeny indicated that *S. chinensis* was composed of clades A and D. Clade D consisted of specimens from Vietnam and Southwest, Central, and Southeast China, corresponding to the *S. c. chinensis* subspecies. Clade A consisted of specimens from Yunnan, Guizhou, and Sichuan, which are the distribution regions of the *S. c. miyiensis* and *S. c. grahami* subspecies; three specimens (YBU 15152, GP 10544, and KIZ 028322) attributed to *S. c. miyiensis* were nested within the other specimens, thereby rendering *S. c. grahami* nonmonophyletic. Therefore, based on priority rule of nomenclature, we concluded that clade A should be represented by *S. c. grahami*, and *S. c. miyiensis* should be considered a synonym of *S. c. grahami*.

Unexpectedly, although clades A and D were highly supported, respectively, these two clades are not sister taxa that together form a monophyletic lineage (Figure 2). The genetic distance between the two clades was 12.6% (cyt. *b*‐based), which exceeds that between certain species (e.g., 9.4% between *S. subpunctatus* and *S. bistrigatus*) (Table 2). In addition, network analyses revealed that the two clades did not share any nDNA haplotype. These findings suggest that both clades represent two distinct species, which means *S. c. grahami* should be elevated to specific rank as *S. grahami*.

Given the taxonomic revision of *S. chinensis* (sensu *lato*), the distribution of *S. grahami* should be redefined accordingly. Thus, based on previous records (Zhao, 2006; Zhao et al., 1998) and our data, we conclude that *S. grahami* is present in southwestern Sichuan, Yunnan, and Guizhou in China (Figure 1).

### 4.2 Distribution of *S. triangularis* and *S. collaris* in China

*Sibynophis triangularis* was originally described based on a single specimen from Thailand (Taylor & Elbel, 1958). Although initially considered a subspecies of *S. collaris*, it was subsequently elevated to a separate species, that is, *S. triangularis* (Taylor, 1965). At present, this species is known to occur in Thailand and Cambodia (Stuart et al., 2006; Uetz et al., 2022; Wallach et al., 2014). However, our molecular phylogenetic reconstruction revealed that a particular sample (FMNH 263023, Cambodia), previously identified as *S. triangularis* (Stuart et al., 2006), formed a well‐supported clade (C) with specimens from Vietnam, Myanmar, and China (southern and northwestern Yunnan). Hence, we confirm that clade C represents the species *S. triangularis*, which is newly reported in China and Myanmar. In China, *S. triangularis* is only known to occur in Fugong and Cangyuan in Yunnan Province.

Our results identified clade B as sister to clade C, representing *S. collaris* based on geographical origin. Unexpectedly, sample CAS 240150 collected from Myanmar and originally identified as *S. collaris* in Chen et al. (2013) formed a distinct mtDNA lineage from clade B, with genetic distances of 8.3% (cyt. *b*‐based) and 7.9% (ND2‐based), respectively (Table 2; Figure 2). These findings suggest that this specimen was misidentified and may represent an undescribed taxon. Thus, further examination of this specimen is required. In addition, in a previous large‐scale systematic study of Chinese snakes, Li et al. (2020) identified one specimen (CHS 244) from Honghe, southern Yunnan, China, as *S. collaris*, and two specimens (CHS 879 and CHS 880) from Motuo, Xizang, China, as *S. chinensis*. Our results showed the first specimen was nested within the clade A and the latter two positioned in the clade B. Thus, we concluded that these samples were misidentified, with the former being *S. grahami* and the latter two being *S. collaris*.

Zhao and Yang (1997), Zhao et al. (1998), Zhao (2006), and Yang and Rao (2008) proposed that *S. collaris* is distributed in southern Xizang and northwestern Yunnan in China. Based on our molecular phylogenetic analyses, however, several specimens collected from northwestern Yunnan, which is geographically close to Motuo, Xizang, were identified as *S. grahami* and S. *triangularis* (Figure 2). Thus, it is most like that *S. collaris* is endemic to Motuo in Xizang, with no occurrence in Yunnan.

## 5 CONCLUSIONS

Based on more extensive sampling, we conducted a comprehensive study of Chinese species of *Sibynophis*, providing new insights into their systematics and distribution. Our results revealed that *S. c. miyiensis* is synonymous with *S. c. grahami*, which should be elevated to a species level. Furthermore, *S. triangularis* is reported as new to China and Myanmar. In total, four species, *S. chinensis*, *S. collaris*, *S. grahami*, and *S. triangularis*, are distributed in China. Consequently, we revised their distribution boundaries in China and re‐examined previously misidentified specimens.

## AUTHOR CONTRIBUTIONS

**Peng Guo:** Conceptualization (lead); data curation (lead); formal analysis (lead); funding acquisition (lead); investigation (lead); methodology (lead); project administration (lead); resources (equal); software (equal); supervision (lead); validation (equal); visualization (equal); writing – original draft (lead); writing – review and editing (lead). **Ping Wang:** Data curation (supporting); methodology (supporting); resources (supporting). **Bing Lyu:** Data curation (supporting); resources (supporting); software (supporting); visualization (supporting). **Qin Liu:** Data curation (supporting); formal analysis (supporting); resources (equal). **Jieyu Zheng:** Data curation (supporting); formal analysis (supporting); resources (supporting). **Chunmei Fu:** Data curation (supporting); formal analysis (supporting); resources (supporting). **Yayong Wu:** Data curation (supporting); resources (supporting). **Guocheng Shu:** Data curation (supporting); resources (supporting). **Shaobing Hou:** Data curation (supporting); resources (supporting).

## FUNDING INFORMATION

This study was supported by the Second Tibetan Plateau Scientific Expedition and Research (STEP) Program (2019QZKK05010105), National Natural Science Foundation of China (NSFC 32000308), and Sciences and Technology Department of Sichuan Province (2020YFSY0033).

## CONFLICT OF INTEREST STATEMENT

The authors declare no conflict of interest.

## Data Availability

All DNA sequences newly generated are deposited in Genbank (accession nos.: OQ981548–OQ981602).
